# Dry age-related macular degeneration classification from optical coherence tomography images based on ensemble deep learning architecture

**DOI:** 10.3389/fmed.2024.1438768

**Published:** 2024-10-09

**Authors:** Jikun Yang, Bin Wu, Jing Wang, Yuanyuan Lu, Zhenbo Zhao, Yuxi Ding, Kaili Tang, Feng Lu, Liwei Ma

**Affiliations:** ^1^Aier Eye Medical Center of Anhui Medical University, Anhui, China; ^2^Shenyang Aier Excellence Eye Hospital, Shenyang, Liaoning, China; ^3^School of automation, Shenyang Aerospace University, Shenyang, Liaoning, China

**Keywords:** dry age-related macular degeneration, optical coherence tomography, ensemble deep learning, NGA, early AMD detection

## Abstract

**Background:**

Dry age-related macular degeneration (AMD) is a retinal disease, which has been the third leading cause of vision loss. But current AMD classification technologies did not focus on the classification of early stage. This study aimed to develop a deep learning architecture to improve the classification accuracy of dry AMD, through the analysis of optical coherence tomography (OCT) images.

**Methods:**

We put forward an ensemble deep learning architecture which integrated four different convolution neural networks including ResNet50, EfficientNetB4, MobileNetV3 and Xception. All networks were pre-trained and fine-tuned. Then diverse convolution neural networks were combined. To classify OCT images, the proposed architecture was trained on the dataset from Shenyang Aier Excellence Hospital. The number of original images was 4,096 from 1,310 patients. After rotation and flipping operations, the dataset consisting of 16,384 retinal OCT images could be established.

**Results:**

Evaluation and comparison obtained from three-fold cross-validation were used to show the advantage of the proposed architecture. Four metrics were applied to compare the performance of each base model. Moreover, different combination strategies were also compared to validate the merit of the proposed architecture. The results demonstrated that the proposed architecture could categorize various stages of AMD. Moreover, the proposed network could improve the classification performance of nascent geographic atrophy (nGA).

**Conclusion:**

In this article, an ensemble deep learning was proposed to classify dry AMD progression stages. The performance of the proposed architecture produced promising classification results which showed its advantage to provide global diagnosis for early AMD screening. The classification performance demonstrated its potential for individualized treatment plans for patients with AMD.

## 1 Introduction

AMD is a retinal disease that is a major cause of blindness around the world (1). According to the World Health Organization, it was estimated that 288 million people globally suffered from intermediate or late-stage AMD (2). As the global population aged, AMD was expected to affect more people. Therefore, it was important to detect and screen AMD, especially for early stage of AMD. Based on the clinical appearance of AMD, it could be classified into early stage, intermediate stage and late stage (3). Early and intermediate AMD, also known as non-advanced dry AMD, were described by a slow progressive dysfunction of the retinal pigment epithelium (RPE) and presence of drusen. The late stage was defined by presence of geographic atrophy (GA) (4).

A recent approval released by the U.S. Food and Drug Administration highlights the importance of early detection of GA, which demonstrated that nGA was a pivotal marker for the prediction of the development of GA (5). It could help clinicians to better detect and screen AMD in the early stage. However, nGA has drusen with a diameter larger than 63 μm without atrophy or neovascular disease. This condition made it difficult to detect nGA accurately. Moreover, complex interference factors of nGA in shape, size and location exacerbated the difficulty to differentiate it from other retinal lesions (6).

**Figure 1:**
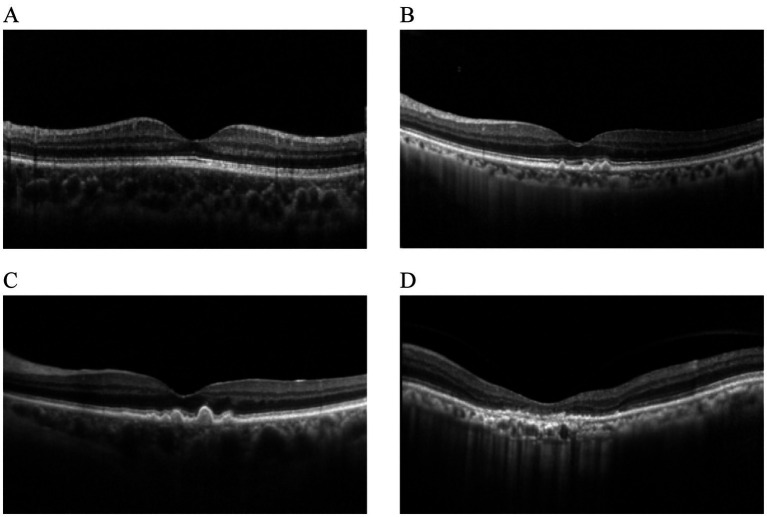
Different stages of dry AMD in OCT imaging. **(A)** Normal. **(B)** Drusen. **(C)** nGA. **(D)** GA.

In traditional retinal images, OCT images enabled visualization of thickness, structure and detail of various layers of the retina (7). In addition, when the retina developed a disease, OCT enabled the visualization of abnormal features and damaged retinal structures (8). Therefore, retinal OCT images were used in this article to detect nGA. In OCT images, spectral-domain OCT features unique in these areas included: subsidence of the outer plexiform layer (OPL) and inner nuclear layer (INL), and development of a hyporeflective wedge-shaped band within the limits of the OPL. These characteristics were defined as nGA, describing features that portended the development of drusen-associated atrophy. Cross-sectional examination of participants with bilateral intermediate AMD revealed that independent risk fact (9). The hypo-reflective wedge in nGA represented the presence of a hyporeflective wedge-shaped band within the limits of the OPL that subsequently developed as the characteristic feature of this stage. There was also typically drusen regression that was accompanied by a vortexlike subsidence of the INL and OPL at this stage. Different stages of dry AMD in OCT images were shown in Figure 1.

Although OCT images were widely applied into the treatment and diagnosis of AMD (10–13), the process was time-consuming due to manual operation and analysis. Ophthalmologists may provide incorrect results even if they had great expertise. With the development of artificial intelligence, machine learning and deep learning algorithms had been used in the diagnosis and treatment tasks, such as classification, detection and segmentation of AMD (14). Deep Learning (DL) had been widely used in the medical field to monitor information in medical images for the diagnosis of various diseases (7, 15–18). Recently, DL integrated with OCT imaging analysis, had been utilized for intelligent and accurate classification of AMD (19). However, most of previous DL-based retinal OCT detection technologies focused primarily on the advanced stage. The main limitation came from the datasets which were mainly comprised of intermediate and late stages of AMD. Additionally, the challenge from the OCT image noise, the accuracy of diagnosis and the division among diverse stages increased the difficulty of early detection (20). A detection architecture based on a two-stage convolution neural network (CNN) with OCT images was proposed by He (21). In the first stage, ResNet50 CNN model was employed to categorize OCT images. Then image feature vector set was accepted by the local outlier factor algorithm in the second stage. This model was tested on the external Duke dataset which consisted of 723 AMD and 1,407 healthy control volumes. This architecture was able to achieve the performance of sensitivity of 95.0% and specificity of 95.0%. Similarly, a two-stage DL architecture was proposed by Motozawa (22). The first stage was capable of distinguishing healthy controls from OCT images. Then AMD with and without exudative changes could be detected in the second stage. This architecture was able to achieve a performance of 98.4% sensitivity, 88.3% specificity and 93.9% accuracy.

Similarly, a visual geometry group CNN architecture was developed by Lee for the categories of retinal diseases (23). This CNN model was trained and tested on 80,839 OCT images to evaluate the performance. The performance of AUC of 92.7% with an accuracy of 87.6% could be obtained. CNN models with fully automated technology were proposed by Derradji to segment retinal atrophy lesions in dry AMD (24). Due to segmentation technologies, this architecture was able to achieve a performance of 85% accuracy and 91% sensitivity.

To better differentiate AMD from healthy controls, Holland developed a pre-trained self-supervised deep learning architecture (25). The performance of 92% AUC was able to be achieved on the test images. However, it was difficult to distinguish between early stage and intermediate stage. To overcome this challenge, Bulut applied Xception models to the detection of AMD based on color fundus images (26). Through analysis of 50 different parameters, this architecture could obtain the highest performance of accuracy of 82.5%. Moreover, Chakravorti proposed an efficient CNN for AMD classification (27). This network trained on fundus images could categorize them in four types of AMD, reducing computational complexity with high performance. Instead of training networks on fundus images, Tomas developed an algorithm for the diagnosis of AMD in retinal OCT images. This algorithms was able to perform the detection of AMD based on the estimate of statistical approaches and randomization (28). Additionally, Zheng extended a five-category intelligent auxiliary diagnosis architecture for common retinal diseases. For the 4 common diseases, the best results of sensitivity, specificity, and F1-scores were 97.12, 99.52 and 98.21%, respectively (29). Vaiyapuri presented a new multi-retinal disease diagnosis model to determine diverse types of retinal diseases. Experimental results demonstrated that this architecture outperformed the exiting technologies for advanced AMD with the performance of accuracy 0.963 (30). Inspired by nature language processing, Lee presented CNN-LSTM and CNN-Transformer. Both deep learning architectures used a Long-Short Term Memory and a Transformer module, respectively with CNN, to capture the sequential information in OCT images for classification tasks. The proposed architecture was superior to the baseline architectures that utilized only single-visit CNN model to predict the risk of late AMD (31). Combining a multi-scale residual convolutional neural network and a vision transformer, Kar featured a generative adversarial network for the detection of AMD (32). Rigorous evaluations on multiple databases validated the architecture’s robustness and efficacy.

In summary, many of the mentioned studies had focused on the application of deep learning and OCT for classifcation of AMD, achieving impressive accuracy rates. However, these studies lacked a comprehensive prediction for nGA. In this paper, we aimed to diagnose early stage of AMD with strong predictor nGA. We provided an ensemble deep learning architecture consisting of four components (ResNet50, EfficientNetB4, MobileNetV3 and Xception) to analyze OCT images. In order to accurately detect the early stage of AMD, an OCT-based ensemble DL architecture was proposed in which the images would be classified into four categories: normal, Drusen, nGA and GA. The main contributions of this work were as follows:

1. To the best of our knowledge, this was the first investigation to use ensemble DL technologies to detect and classify nGA.
2. The proposed architecture showed its advantage and provided detection results which could be utilized as a useful computer-aided diagnostic tool for clinical OCT-based early AMD diagnosis.
3. This paper proposed an ensemble technique by combining the predictions of four base CNNs—ResNet50, EfficientNetB4, MobileNetV3 and Xception. Based on the knowledge gained from ImageNet dataset, each base CNN was fine-tuned for the specific OCT image classification task.

## 2 Methods

### 2.1 Datasets

Although there were some public OCT datasets, they were not suitable for the detection of early stage of AMD. This study was retrospective. We used OCT images collected in 2019–2023 which were gathered from 1,310 patients (male and female) of diverse age groups and ethnicity from Shenyang Aier Excellence Eye Hospital. The images in this dataset had been divided into four different classes: normal, drusen, nGA and GA. The training set and the test set were about 80 and 20% of the patients, respectively. All OCT images were captured from Heidelberg Spectralis HRA which was able to provide 6 mm × 6 mm B-scan length. The quality of OCT images were analyzed by ophthalmologists. All OCT images were clear and free of artifacts. Every OCT image was either normal or AMD without other retinal diseases. According to the judgment of ophthalmologists, OCT images that met the selection criteria were stored in the database. Any participant with any other ocular, systemic or neurological disease that could have an impact on the assessment of the retina, was excluded.

To improve the generalizability and reduce the risk of overfitting of the proposed architecture, this paper employed three-fold cross-validation to evaluate the performance. In one epoch of cross-validation, two-fold OCT images were used for training while the rest of OCT images were used for test. The training and test process would be performed three times and the average of results could be utilized to assess the performance of the proposed architecture. The number of training and test images were detailed in Table 1.

**Table 1:** The detail of three cross-fold training and test datasets.

OCT Datasets	Fold 1		Fold 2		Fold 3	
	Train	Test	Train	Test	Train	Test
Patients (%)	1,040 (80%)	270 (20%)	1,040 (79%)	270 (21%)	1,040 (80%)	270 (20%)
Images (16384)	13,107	3,277	12,943	3,441	13,107	3,277
Normal (3072)	2,455	617	2,427	645	2,455	617
Drusen (5120)	4,094	1,024	4,045	1,075	4,094	1,024
nGA (4096)	3,277	819	3,236	860	3,277	819
GA (4096)	3,277	819	3,236	860	3,277	819

### 2.2 Image enhancement

**Figure 2:**
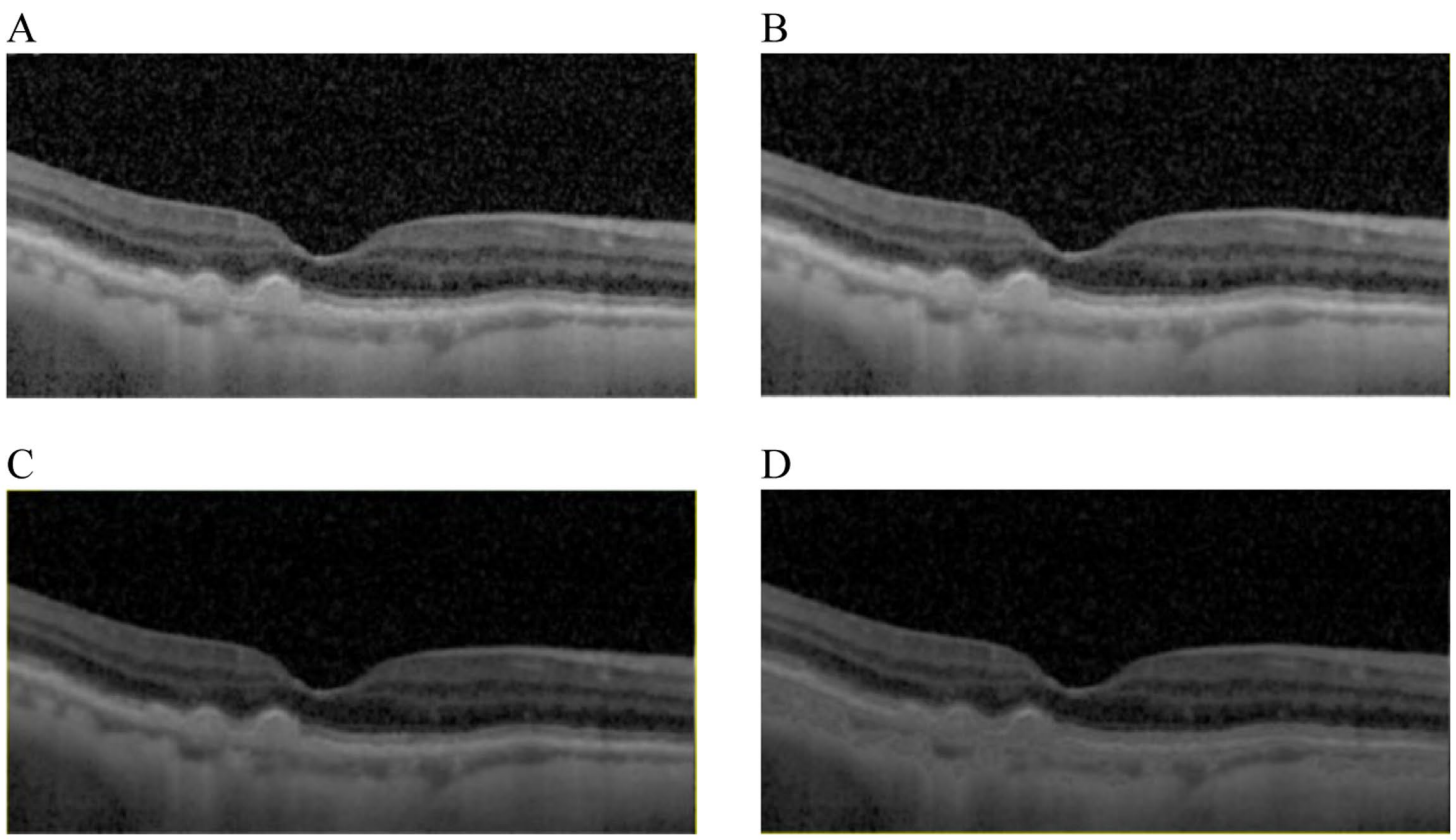
Results comparison from image enhancement. **(A)** The original image. **(B)** The OCT image from **(A)** with diffusion filtering. **(C)** The OCT image from **(B)** with linear enhancement. **(D)** The OCT image from **(C)** with the exponential enhancement.

The aim of OCT images enhancement was to provide high quality images which would improve the performance of the proposed architecture. The visibility of significant features would be enhanced by image enhancement algorithms, such as diffusion filtering, linear enhancement and exponential enhancement. Results of different image enhancement algorithms were displayed in Figure 2, where the original OCT image was shown in Figure 2A. To start with, a diffusion filtering algorithm (33) was applied to reduce noise from the original OCT image, as presented in Figure 2B. Then linear enhancement (34) was employed to highlight the contrast between background and retinal layers, as shown in Figure 2C. At last, the OCT image was processed based on exponential enhancement (35) to further accentuate contrast between different layers. With the enhancement procedure, the final result could be obtained, as shown in Figure 2D.

### 2.3 Ensemble deep learning architecture

**Figure 3:**
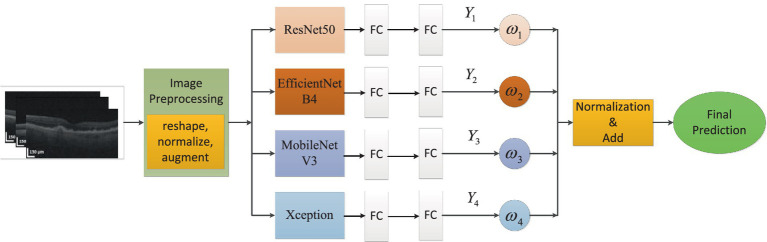
The global ensemble architecture.

In this article, we built an ensemble deep learning architecture which consisted of four base models (ResNet50, EfficientNetB4, MobileNetV3 and Xception). After image enhancement, OCT images were further processed via image preprocessing, such as reshaping, normalization and augmentation. Then the OCT images were fed to every base model which was pre-trained on ImageNet and connected to full-connection layers. The prediction scores $Y_{i}$ (*i* = 1,2,3,4) were obtained from four models. The weights would also be calculated based on these scores. As distributing weights to base models, we could obtain the final prediction through adding and normalizing these prediction scores. The global ensemble architecture was presented in Figure 3.

**Figure 4:**
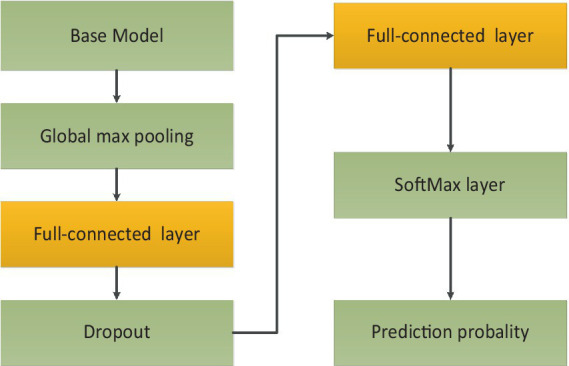
The training process of transfer learning.

In the OCT image pre-processing step, all OCT images were converted into gray values which ranged from 0 to 1. In order to obtain optimal classification performance, we tested OCT images with different shapes. We found that input images with 320 × 320 could provide the best performance. The visibility of AMD features were emphasized to accentuate contrast between different retinal layers. Moreover, data augmentation could improve the generality of the proposed method. The size of training data could be augmented. The number of original images was 4,096. Every original image was rotated $90^{\circ }$ , $180^{\circ }$ , and $270^{\circ }$ respectively. Besides all original images could be flipped. After augmentation, the total number of images was 16,384. 80% of the total images were used for training and the unseen images were employed for test purpose. The whole dataset was labeled by two ophthalmologists. Then the proposed architecture was comprised of four fine-tuning models. To reduce training time, transfer learning technology was used. The base models were pre-trained on ImageNet dataset. The weights before ‘FC’ were kept frozen. Then the training process would fine-tune weights between ‘FC’ layers with a learning rate 0.001. To further avoid overfitting and reduce computation, ‘dropout layer’ with a dropout rate of 0.4 had also been added between ‘FC’ layers. Because classification of AMD was performed with four categories, soft-max activation with four categories had been added after ‘FC’ layer for classification task of AMD. The training process of transfer learning was shown in Figure 4.

**Figure 5:**
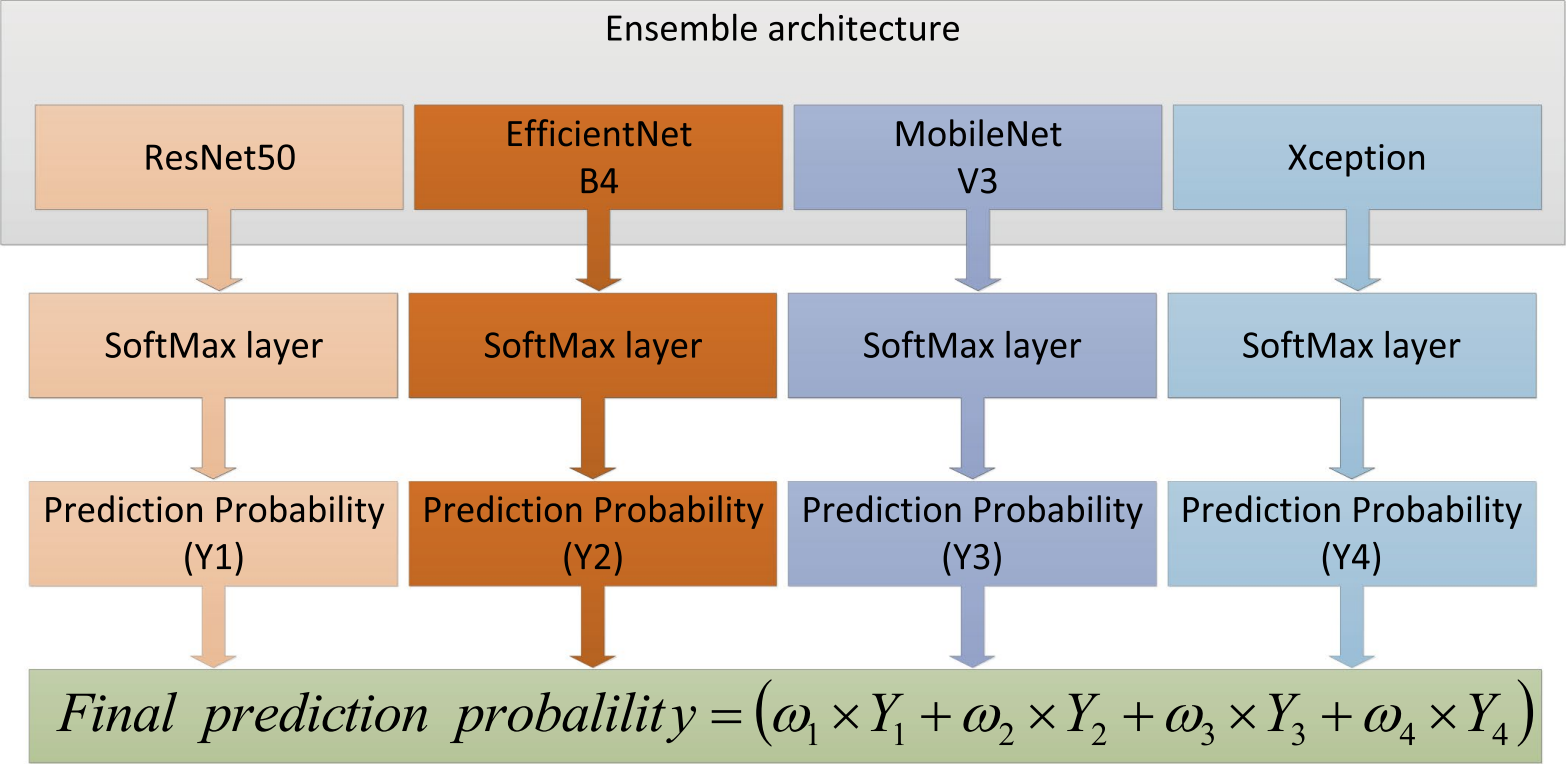
The combination strategy of the proposed ensemble-based architecture.

The proposed architecture could be formed with the following steps: First, different base models, also known as CNNs, were analyzed after fine-tuning. Based on the performance of different base models, it could be found that the ResNet50, EfficientNetB4, MobileNetV3 and Xception had better performance compared to other CNN models on the test dataset. Then a comparative analysis of the weights combination strategies among different base models were performed. These strategies contained simple averaging, weighting function, majority voting and stacking methods. The weighting function was proportional to the performance of base models on test dataset. From these comparison results, the weighting function strategy could obtain the best performance for the classification of early AMD. The combination strategy of the proposed ensemble-based architecture was shown in Figure 5.

It could be found that four base models produced four prediction scores $Y_{i}$ (*i* = 1,2,3,4). The final prediction score could be calculated based on weights which were proportional to the performance of base models. At last, the accuracy of diagnosis was compared with the ground truth to evaluate the performance of the ensemble deep learning architecture. The weights could be calculated based on the following mathematical formulation, as shown in Equation 1:


(1) $$\omega =F_{com}\left(Y_{1}\left(x\right),Y_{2}\left(x\right),Y_{3}\left(x\right),Y_{4}\left(x\right)\right)$$

Where $F_{com}$ was the combination function which represented an aggregation strategy with various weights ω . If the the prediction probabilities from i th model was $Y_{i}$ , then the weight $\omega_{i}$ could be expressed as Equation 2:


(2) $$\omega_{i}=\frac{Y_{i}}{\overset{4}{\underset{n=1}{\sum }}Y_{n}}$$

The weights which denoted the significance of every base model. The final prediction probability *P* on the test dataset could be obtained based on the weights combination strategy, as shown in Equation 3:


(3) $$P=\overset{4}{\underset{i=1}{\sum }}\omega_{i}Y_{i}$$

The experimental results would be obtained and analyzed based on the proposed architecture in the next section.

## 3 Results

All experiments were conducted in Pytorch and the hardware was composed of 64 hyper-thread processors, 8 × RTX 2080 Ti, and windows10. All OCT images were set to the shape 320×320 .

Base CNN models were evaluated on the test dataset. The classification task was performed for four categories of dry AMD (normal, drusen, nGA, and GA). Diverse metrics were used to evaluate the efficacy of base models, including accuracy (Acc), sensitivity (Sen), specificity (Spc) and F_1_-score (F_1_) for overall classification performance. Sen described how well the test caught all of positive cases and Spc described how well the test classified negative cases as negatives. F_1_ was a metric that offered an overall measure of the model’s accuracy. These metrics could be expressed from Equations 4–7:


(4) $$\mathrm{Accuracy}\left(Acc\right)=\frac{TP+TN}{TP+FP+TN+FN}$$

(5) $$\mathrm{Sensitivity}\left(Sen\right)=\frac{TP}{TP+FN}$$

(6) $$\mathrm{Specificity}\left(Spc\right)=\frac{TN}{TN+FP}$$

(7) $$F_{1}-\mathrm{score}\left(F_{1}\right)=\frac{2TP}{2TP+FP+FN}$$

Where TP , TN , FP , and FN represented true positives, true negatives, false positives, false negatives, respectively. The performance of each base model and the proposed architecture would be compared and evaluated using above performance metrics. 80% of the sample images were employed for training and the rest of images were used for testing. Different base models were assessed on the test dataset. The performance results were presented in Table 2.

**Table 2:** The performance comparison among different methods.

Methods	Sen (%)			Spc (%)			F_1_ (%)			F_1_ (average)
	Fold1	Fold2	Fold3	Fold1	Fold2	Fold3	Fold1	Fold2	Fold3	
ResNet50	92.67	92.16	91.45	93.52	93.33	92.95	92.82	93.29	92.17	92.76
EfficientNetB4	90.23	90.15	91.06	92.44	91.22	92.15	92.63	91.29	91.18	91.71
MobileNetV3	92.54	90.33	93.08	93.11	87.15	91.22	92.25	89.93	92.54	91.57
Xception	92.71	89.66	92.13	91.08	90.53	84.57	91.12	91.56	90.81	91.16
VGG19	90.33	92.58	90.87	91.54	91.25	91.53	91.85	89.92	87.75	89.84
InceptionResNetV2	92.31	87.88	90.21	89.66	90.18	89.55	89.48	89.76	89.73	89.66
EfficientNetB0	90.55	90.28	88.93	84.57	91.16	90.15	88.61	89.28	87.63	88.51
NASNetMobile	91.56	89.15	90.23	86.43	90.22	87.38	87.33	88.07	89.06	88.15

Different ensemble strategies were also compared, such as majority voting, stacking, simple averaging, and weighting function (the proposed method). Majority voting meant that the prediction result from every base model was defined as a “vote.” The most votes were used as the final prediction result. Stacking could be conducted by training classifiers on the combined classification scores in an ensemble architecture. Then the ensemble architecture would classify test images based on the trained classifiers. Simple averaging overlooked the effect from weights. It used an average weight to process every prediction scores. Instead of obtaining an average weight, the weighting function would allocate weights to various base models. The weights were proportional to the performance of base models on the train dataset. The prediction scores would be further processed to get the final prediction result. The comparison results were detailed in Table 3.

**Table 3:** The performance comparison from different ensembling strategies.

Strategy	Acc (%)	Sen (%)	Spc (%)	F1 (%)
majority voting	93.15	88.56	92.54	92.37
stacking	94.32	95.49	86.25	92.22
simple averaging	90.11	88.23	89.25	84.33
weighting function	96.51	93.31	94.56	97.45

**Figure 6:**
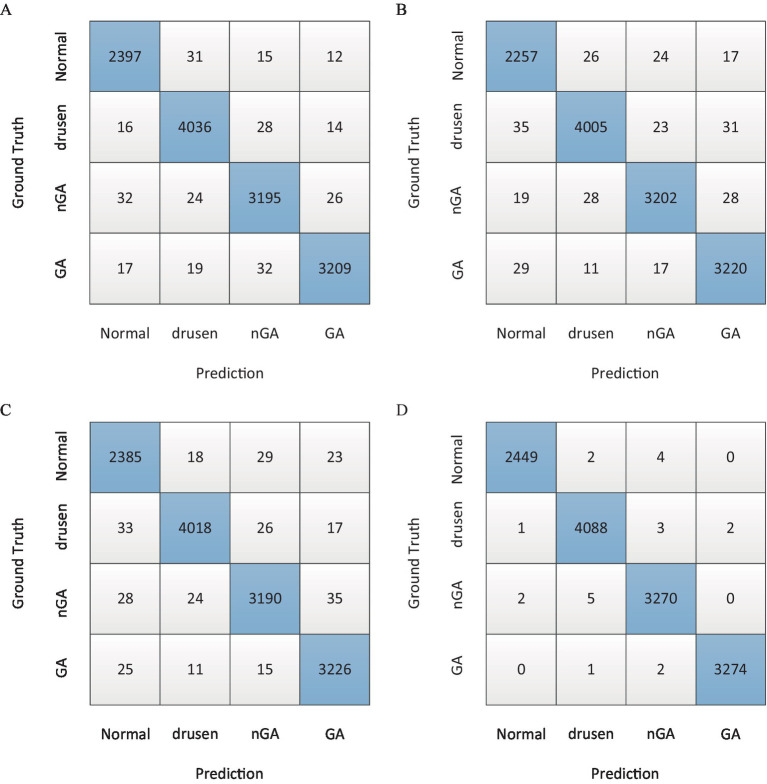
The confusion matrix of overall classification results. **(A)** Majority voting. **(B)** Stacking. **(C)** Simple averaging. **(D)** Weighting function.

Based on the F_1_ score in Table 3, the confusion matrix was also utilized to further show overall classification results with different architectures, as shown in Figure 6.

**Figure 7:**
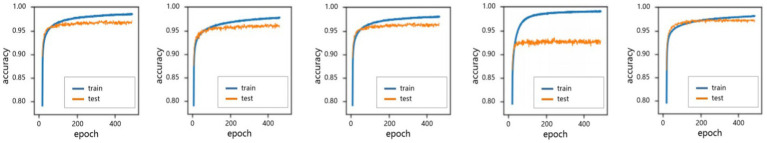
The performance of accuracy in 400 epochs. **(A)** ResNet 50. **(B)** EfficientNetB4. **(C)** MobileNetV3. **(D)** Xception. **(E)** Proposed.

The performance between the proposed ensemble architecture and base models were also analyzed. The training epoch was set to 400. In every epoch, the corresponding results were recorded. The comparison results were shown in Figure 7. Four categories of dry AMD were used to analyze the performance of classification. The classification Acc from base models and ensemble architecture were presented in Table 4.

**Table 4:** The Acc (%) comparison among different classification results.

	ResNet50	EfficientNetB4	MobileNetV3	Xception	Proposed
Normal	92.66	93.25	88.64	91.65	96.66
Drusen	92.71	91.64	92.57	87.30	94.85
nGA	91.56	89.66	89.43	85.22	98.21
GA	92.55	93.85	89.64	92.74	96.31
F_1_ (average)	92.82	91.71	91.57	91.16	97.45

**Figure 8:**
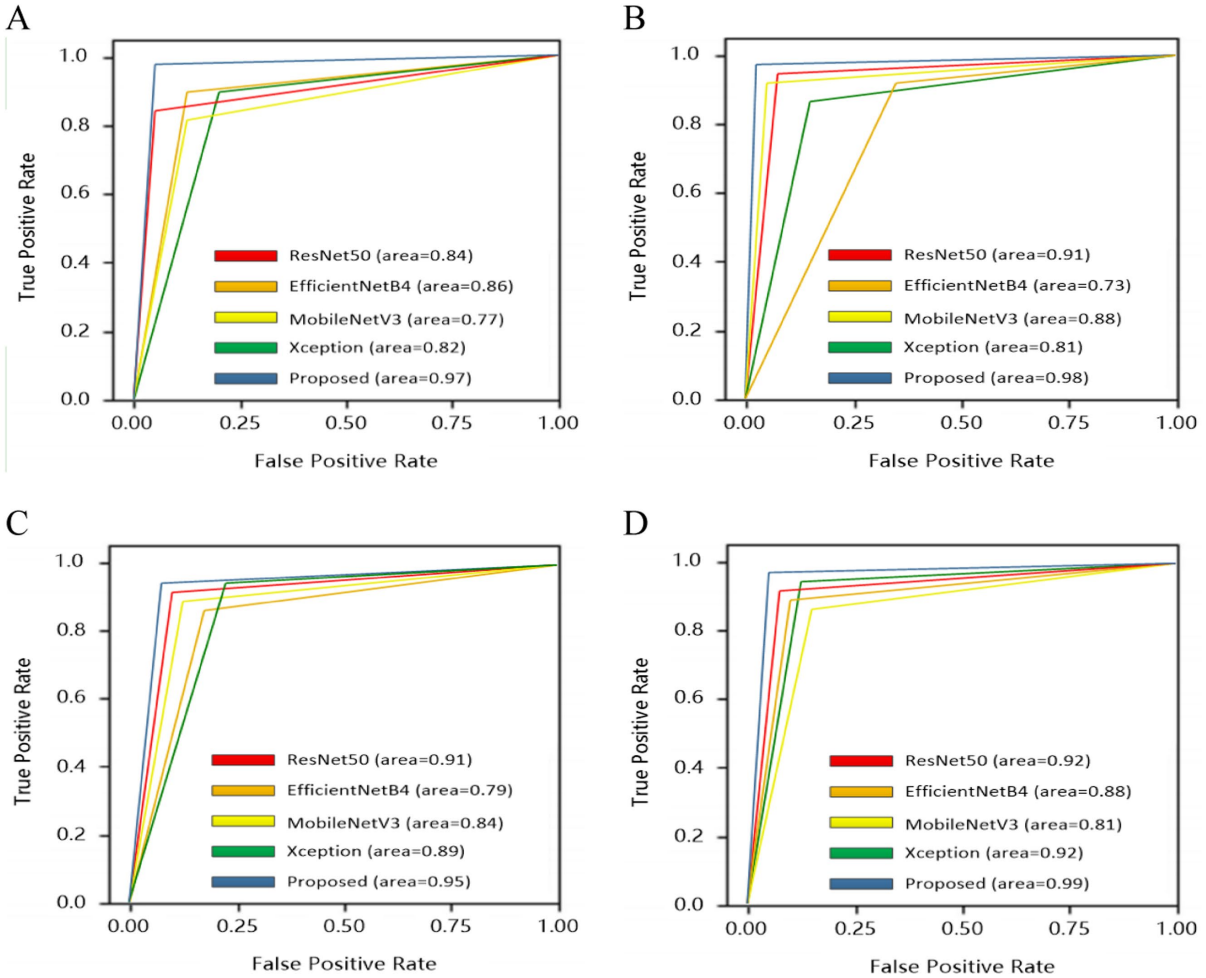
ROC comparison among different methods. **(A)** Normal. **(B)** Drusen. **(C)** nGA. **(D)** GA.

To give a comprehensive analysis from true positive rate and false positive rate, receiver operating characteristics curve (ROC) was also plotted for four categories: normal, drusen, nGA and GA, as shown in Figure 8.

**Figure 9:**
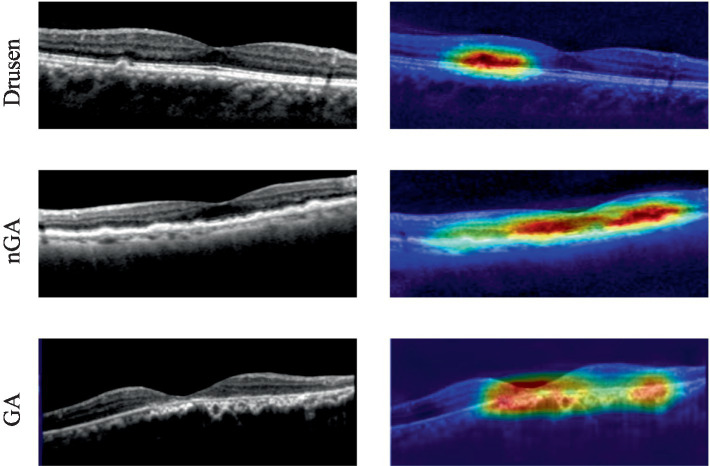
Heatmaps from dry AMD with pathological features.

The visualization of heatmaps could also be generated to improve the interpretability in OCT images based on Grad-CAM. To show the classification basis, heatmaps of drusen, nGA, GA were generated respectively, as shown in Figure 9.

## 4 Discussion

In this study, an ensemble deep learning architecture was proposed. To choose base models, different base models were tested and the performance results were shown in Table 2. The results were sorted in descending order based on F_1_ score since it offered a comprehensive evaluation of different models. Therefore F_1_ score could provide a basis for base models selection. Notably, four CNN models (ResNet50, EfficientNetB4, MobileNetV3 and Xception) could produce better results due to the consideration of local detail features and global semantic features. These base models would be served as base components in the ensemble architecture.

For the same base models, there were different combination strategies. In this study, majority voting, stacking, simple averaging and weighting function (the proposed method) were compared. The comparison results in Table 3 showed that both stacking and weighting function had better accuracy with 94.32% Acc and 96.51% Acc, respectively. Stacking strategy had the similar performance of sensitivity to the weighting function strategy which had better performance with 94.56% Spc. As presented in the column of F_1_ score, it could be found that weighting function had the best performance of classification with 97.45% F_1_ score. Moreover, the confusion matrices in Figure 6 demonstrated the advantage of the proposed architecture which could provide the best overall classification with less errors.

All base models could be fused based on tasks. Therefore, four categories of dry AMD were used to analyze the performance of different models. Comparison results were shown in Figure 7. It could be found that the proposed architecture and base models had similar performance. There were no over-fitting. Besides, the proposed architecture had better performance than base models with training epochs increasing. From comparison results in Table 4, it could also be found that the proposed architecture could generate commendable results. Compared with base models, the ensemble architecture could significantly improve the classification performance with the highest accuracy for all classes, especially for nGA. In terms of sensitivity and specificity, the proposed architecture outperformed all base models. It demonstrated that the proposed architecture could detect true positives and true negatives much better. For base models, F_1_-score were 92.82, 91.71, 91.57, and 91.16%, respectively, while the proposed method could archive the highest F_1_-score (97.45%). The comparison results demonstrated that the proposed architecture had better robustness and better performance of accuracy due to the combination of base models. ROC was provided to analyze overall performance of classification. Comparison results in Figure 8 showed that the maximum area could be obtained from the proposed architecture. It meant that the proposed architecture could provide more accurate classification results, especially for nGA.

The heat maps generated through Gram-CAM validated that typical features from dry AMD could be successfully detected. Three categories of pathological features (drusen, nGA and GA) could be correctly accentuated. In particular, the early stage of AMD with nGA could be correctly highlighted and observed, as shown in Figure 9. The heat maps demonstrated that the proposed architecture could successfully identify distinctive features and relevant lesions.

## 5 Conclusion

The intention of this article was to provide an architecture for an automated diagnosis and classification of AMD using OCT images, including the detection of early-stage dry AMD with nGA. The proposed architecture did not need to segment biomarkers. By combining image enhancement and base CNN models, the performance of detection of dry AMD could be improved. Experimental results showed that three categories of pathological features could be correctly detected and observed, particularly for the nGA feature. The proposed ensemble architecture and base models had similar performance. There were no over-fitting. Moreover, the proposed ensemble architecture had the best classification performance for the present OCT images classification task. It suggested that the proposed ensemble architecture was superior in classification task for early stage of AMD. In the future, multi-modal images such as fundus photography and angiography can be used to supplement OCT images. Besides the diagnostic performance can be improved by integrating other artificial intelligence technologies such as segmentation and attention mechanism.

## Data Availability

The raw data supporting the conclusions of this article will be made available by the authors, without undue reservation.
